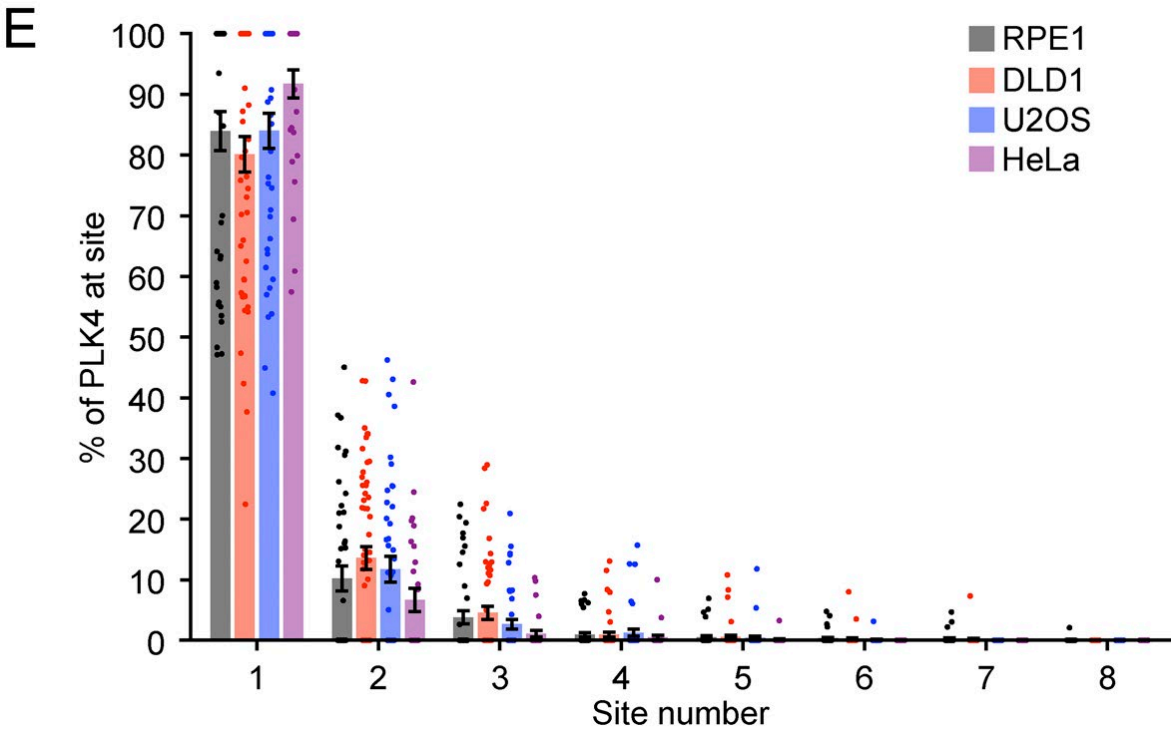# Correction: PLK4 self-phosphorylation drives the selection of a single site for procentriole assembly

**DOI:** 10.1083/jcb.20230106912112023c

**Published:** 2023-12-14

**Authors:** Phillip Scott, Ana Curinha, Colin Gliech, Andrew J. Holland

Vol. 222, No. 12 | https://doi.org/10.1083/jcb.202301069 | September 29, 2023

After publication, the authors discovered that the x-axis labels in Fig. 1 E were misaligned relative to the dataset in the graph. The corrected panel is shown here and has been replaced in the paper.

The conclusions of the paper are not affected by this error, and all the plotted data and discussion of the data presented remain correct.

This error appears in the print and any PDF downloaded on or before December 12, 2023. The authors apologize for any confusion this may have caused.

**Figure 1:**